# Supplementary material for: SARS-CoV-2 detection in setting of viral swabs scarcity: Are MRSA swabs and viral swabs equivalent?
Source: PLoS One. 2020 Aug 5;15(8):e0237127. doi: 10.1371/journal.pone.0237127 (PMC7406068; doi:10.1371/journal.pone.0237127)
Supplement: Attachment — Submitted filename: Response to Reviewers PLOS ONE.docx [file pone.0237127.s002.docx]

Response to reviewers:

I would like to thank PLOS ONE and its reviewers for their prompt and inciteful review of our manuscript, PONE-D-20-14345.

With regards to the reviewers’ comments, our newest submission includes the following changes:

1. Regarding the comment that we need to emphasize that our study was limited by its small size, we have added this sentence to the limitations, “Our sample size is both small and from one center, and needs to be confirmed in future larger studies including multiple centers with multiple technicians obtaining samples.”
2. We will make our Data available once we deidentify it. It can be obtained by e-mailing the corresponding author.
3. Our work was previously published online and without peer review with MedRxiv.
4. I have an ORCID ID: 0001-9253-8630 and will upload it with my submission.
5. I have inserted “(Figure 1)” in the appropriate area of the body of the text.
6. Regarding Reviewer 1’s specific comments:
   a. Please see comment 1 regarding a change to our limitations.
   b. Please see comment 1 regarding the comment about technician-specific variability.
   c. A new paragraph was added to the Discussion to summarize a new J Clin Microbiol paper by Rogers et al, who measured the stability of SARS-CoV-2 RNA in liquid Amies, which is what the reviewer stated should be done. Rogers et al found that the RNA was stable out to at least 7 days at room temperature and at least two weeks at refrigerator temperature. There was some loss of RNA after two weeks while frozen, but not enough to change their result interpretations. Their study was nicely complementary to ours and reinforced our findings regarding the suitability of ESwabs plus liquid Amies for COVID-19 testing.
   d. I have added additional references in the introduction as requested.
   e. We changed the first sentence in the Results section to read, “We sampled 81 unique patients, of which the viral swabs were positive for SARS-CoV-2 PCR on the DiaSorin test in 19.”
   f. We removed the sentence in the limitations about our study being conducted only in Veterans and maybe not being generalizable.
   g. We changed our “short and incomplete” sentence to now read, “ The viral swab specimens were nasopharyngeal; each MRSA swab specimen was collected by accessing a single nare, and extending the swab as far into the mid-turbinate area as the thickness of the swab would allow.”
7. Regarding Reviewer 2’s specific comments:
   a. A brief description was added to the Methods section regarding the primers and probes used in the Diasorin Simplexa assay, and the region of the viral genome that they target. Please note that the sequences of the primers and probes are not available. They are not provided in the manufacturer's Instructions for Use available on the FDA EUA website, nor have they published elsewhere as far as we can determine. It is likely that the manufacturer considers the sequences proprietary information. However, the sequences per se are not actually germane to our study, so this doesn’t really matter.

We would like to thank you for your suggestions for improvement. We feel that by responding to them appropriately, our manuscript is much stronger. Thank you again.

Sincerely,

Dan Federman, MD
